# Recipe for High Blood Pressure: Synergistic Effects of Stress and Lead

**DOI:** 10.1289/ehp.115-a417a

**Published:** 2007-08

**Authors:** Valerie J. Brown

Human research has shown associations between lead exposure and hypertension as well as between stress and hypertension. A new study now shows for the first time that stress amplifies the effects of lead exposure on blood pressure in humans **[*EHP* 115:1154–1159; Peters et al.]**.

National Heart, Lung, and Blood Institute guidelines define high blood pressure as systolic pressure over 140 mmHg or diastolic pressure over 80 mmHg. Systolic pressure tends to rise with age whereas diastolic pressure tends to decline. High readings of either type significantly raise the risk of stroke and coronary disease.

A multi-institutional team examined data from 513 participants in the Normative Aging Study, a longitudinal study of men in the greater Boston area begun in 1963. Using data from the period 1987–1996, the researchers compared blood pressure status with self-reported stress levels (determined by questionnaires) and body burden of lead (determined by bone lead tests). About half the participants did not have hypertension; for this group the researchers analyzed follow-up data until 2004 or the participants developed hypertension, whichever came first. In the latter group, 97 new cases of hypertension were observed.

The study participants averaged 66.9 years of age. This put them in the age group most likely to have high systolic pressure, and meant they were old enough to have been exposed to significant amounts of lead before public policy changes in the 1970s reduced environmental lead from gasoline, paint, and other sources.

After accounting for other known hypertension risk factors, including age, body mass index, family history, and alcohol consumption, the researchers found that the effect of lead was “most pronounced among highly stressed individuals, independent of demographic and behavioral risk factors.” Among those reporting high stress, the risk of developing hypertension was more than 2.5 times that of participants reporting low stress for each standard deviation increase in bone lead. The current study was consistent with previous research suggesting that lead and stress affect only systolic pressure.

The authors note that their study does not address lead’s potential effects at various life stages. For example, early exposure leading to neurological damage might make people more likely to experience events as stressful. In addition, the study was limited in that participants were all male, were 97% white, and had higher than median incomes. Given that both lead exposure and stress tend to be elevated in lower socioeconomic strata, their effects on blood pressure may be more serious in those populations.

